# Health of midlife and older adults in China: the role of regional economic development, inequality, and institutional setting

**DOI:** 10.1007/s00038-017-0970-9

**Published:** 2017-04-22

**Authors:** Xuejie Ding, Francesco C. Billari, Stuart Gietel-Basten

**Affiliations:** 1 Department of Sociology, University of Oxford, Oxford, UK; 2 Carlo F. Dondena Centre for Research on Social Dynamics and Public Policies and Department of Policy Analysis and Public Management, Bocconi University, Milan, Italy; 3 Department of Social Policy and Intervention, University of Oxford, Oxford, UK

**Keywords:** Biomarkers, China, Economic development, Health-related public infrastructure, Income inequality, Midlife and older adults

## Abstract

**Objectives:**

To document the association between economic development, income inequality, and health-related public infrastructure, and health outcomes among Chinese adults in midlife and older age.

**Methods:**

We use a series of multi-level regression models with individual-level baseline data from the China Health and Retirement Longitudinal Survey (CHARLS). Provincial-level data are obtained both from official statistics and from CHARLS itself. Multi-level models are estimated with different subjective and objective health outcomes.

**Results:**

Economic growth is associated with better self-rated health, but also with obesity. Better health infrastructure tends to be negatively associated with health outcomes, indicating the likely presence of reverse causality. No supportive evidence is found for the hypothesis that income inequality leads to worse health outcomes.

**Conclusions:**

Our study shows that on top of individual characteristics, provincial variations in economic development, income inequality, and health infrastructure are associated with a range of health outcomes for Chinese midlife and older adults. Economic development in China might also bring adverse health outcomes for this age group; as such specific policy responses need to be developed.

**Electronic supplementary material:**

The online version of this article (doi:10.1007/s00038-017-0970-9) contains supplementary material, which is available to authorized users.

## Introduction

Three overarching ideas have been proposed to understand health inequality across time and place. First, a higher level of economic development is seen as leading to better health outcomes (Preston 1975). Second, in a thesis that has been widely discussed, greater income inequality is seen to worsen health outcomes, through both psychosocial and material mechanisms (Wilkinson and Pickett 2010). Third, the provision of health services and infrastructure is considered a key determinant of health (Anand and Ravallion 1993), which may even moderate the effect of economic development and income inequality.

It is widely acknowledged that economic development allows to improve the quality of goods and services such as food, health care, and medical services, which in turn leads to improved health and nutrition among the population (Anand and Ravallion 1993). However, recent research shows that economic growth might also foster the prevalence of chronic diseases, such as obesity, diabetes and their complications. Evidence shows that the urbanisation and economic progress in China have led to a radical reduction in overall and occupational physical activity (Ng et al. 2009; Sherif and Sumpio 2015), as well as an increase in fat consumption.

The link between income inequality and health is also controversial, both at the theoretical level and empirically. Wilkinson and Pickett (2010) suggest that individuals who live in a more equal society have on average better health outcomes than their counterparts living in less equal societies. This idea, formulated with specific reference to developed societies, is hypothesised to work through both psychosocial and material mechanisms. More specifically, income inequality would influence health through relative deprivation. For individuals with relatively low incomes, inequality generates negative emotions such as shame and stress that harm health through “psycho-neuro-endocrine” mechanisms, and induces unhealthy behaviours such as smoking and alcohol abuse. Relative deprivation also impairs health for the whole population, through a reduction of social capital and social cohesion: in more unequal societies, participation in community activities is reduced, and trust and efficacy within a community are weakened, with potential effects on the health of the entire population, including those higher up the income range (Kawachi and Kennedy 1999). The evidence from the large body of empirical work testing the income inequality hypothesis is however mixed. While Pickett and Wilkinson (2015) insist on the relationship between inequality and population health being causal, methodological concerns and empirical evidence have cast doubts on the causal nature of this relationship. Studies using large and high-quality data sets found that the effects of income inequality, which are significant in OLS regression models, tend to disappear or become markedly weaker when more appropriate fixed-effect or multi-level regression models are used (Beckfield 2004). Mellor and Milyo (2003), in a study on the US, added a variety of controls to their analysis and found that income inequality has no significant effect on health. Biggs et al. (2010) and Rajan et al. (2013) revisited the income inequality hypothesis in Latin America and India and found no evidence supporting the claim that income inequality is detrimental to health in these less developed global settings.

The provision of health-related public infrastructure [HRPI] has been shown to make a difference to health outcomes through its link to economic development and income inequality. Higher levels of economic development allow greater public investments in HRPI at the aggregate-level, and make out-of-pocket expenditures in medicines and services at the individual-level more affordable (Dollar and Kraay 2002). Societies with higher levels of income inequality, with the US as a textbook case, tend to spend less on improving public health or human capital development. This lower level of public investment weakens the opportunities that individuals have to improve their living standards and health (Kentikelenis et al. 2015). Improved health services and infrastructures make healthcare more affordable and accessible, with the potential to buffer the negative effects arising from income inequality (Ellwardt et al. 2014).

In summary, the literature emphasizes three interdependent aggregate-level determinants of health: economic development, income inequality and investment in health infrastructure. Although it has been widely recognized that individual characteristics explain most of the variation in health disparity, this study aims to understand the effects of these contextual factors for a specific group: Chinese midlife and older adults (age 45 and above). In China, the rapidly growing elderly population poses great challenges for global health. Over the past few decades, the country has experienced unprecedented economic growth, accompanied by dramatic increase in income inequality (Li and Zhu 2006). Although a high rate of improvement in HRPI has occurred in China since the 1990s (Liu 2004), the ever-increasing share of personal health and medical expenditure still poses a challenge for the government (Zhang and Kanbur 2005). It is hence paramount to examine whether the effects of economic development on health are modified by inequality or health infrastructure.

We specifically focus on midlife and older adults. Midlife is a period in life in which limited physical functioning and manifest chronic diseases start to become widespread. Furthermore, this period of life is linked to growing healthcare needs, which increase burdens on existing formal and informal health infrastructures (Muramatsu 2003). Midlife and older adults are therefore vulnerable to socio-economic disadvantages and changes in their living environment, and are particularly relevant to understanding the relationship between economic growth, inequality and medical infrastructure on health (Mosquera et al. 2016).

Previous studies of the link between wealth/inequality and health link have relied on survey data, or hospital records. While our study also uses self-rated health (SRH), we additionally employ biomarker data to measure health risks. The utilization of a series of biomarkers provides some attractive features. Biomarkers are measured objectively, minimizing systematic reporting errors caused by bias and preferences. They provide an identification of pre-disease pathways, since physiological processes are often below the individual’s threshold of perception. We also include allostatic load (AL)—a comprehensive index that incorporates multiple biomarker risk factors, which is expected to better predict future health risks than any single risk factor by itself. However, the mechanisms underlying the links between income inequality and specific biomarkers are not yet clear. Any (or all) of the aforementioned mechanisms could mediate the possible relation between income inequality and biomarkers in cardiovascular diseases and physical functions. “Psycho-neuro-endocrine” factors could potentially affect diet choice and rates of physical activity, consequently affecting blood pressure, inflammation and grip strength. In addition, less egalitarian provinces may invest less resources in promoting and maintaining healthy behaviours, and may also invest less in health education and preventive care. The exposure to economic underdevelopment and income inequality accumulates throughout the life course, and places demands on the biological system (e.g. cardiovascular and metabolic system), ultimately leading to greater system dysregulation, subsequently enhancing risk for poor health and functioning (Gruenewald et al. 2012). The prevention, diagnosis, and long-term treatment of non-communicable and chronic diseases as well as physical functioning indicated by biomarkers are heavily relying on health infrastructures (Blankart 2012). Some recent studies focusing on the United States found that risk factors for cardiovascular diseases, including hypertension, diabetes, and grip strength, show variation at regional levels and are associated with state-level income inequality (Cushman et al. 2009; Diez-Roux et al. 2000; De Vries et al. 2014). To our knowledge, this paper is the first to study the associations between income inequality, economic development, health infrastructure and health as measured through biomarkers among Chinese midlife and older adults.

## Methods

### Data

Given our multi-level framework, we need data both at the individual and aggregate (provincial) level. Individual-level SRH and physical measures of girth, blood pressure, handgrip strength, and covariates comes from the 2011 national baseline survey of the Chinese Health and Retirement Longitudinal Study (CHARLS). CHARLS, modelled after the US Health and Retirement Study, provides a wide range of information, from socio-economic status and social support to health conditions for a randomly selected and nationally representative sample of Chinese residents age 45 and above, living in households. The total analytical sample of the baseline national wave comprises 17,368 individuals across 28 provinces. Among the interviewed subjects, 11,635 individuals (67%) accepted to provide a blood sample, and therefore allow us to have biomarker information on glycosylated haemoglobin, triglycerides, cholesterol ratio and C-reactive protein. Since women had a slight higher blood response rate than men (69 versus 65%), we use the sample weights that the CHARLS team has calculated to correct for household and individual non-response as well as non-participation in the blood collection (for details, see Zhao et al. 2014).

### Outcome variables

We study SRH, seven single biomarkers, and AL as outcomes. SRH and single biomarkers are coded as dichotomous indicators. AL is treated as continuous variable. The seven single biomarkers are: girth, glycosylated haemoglobin, blood pressure, triglycerides, cholesterol ratio, C-reactive protein, and grip strength. These biomarkers are good predictors of diseases. For instance, Ridker et al. (2003) found that C-reactive protein helps predict risk of heart attack and stroke. Abdominal girth (Alberti and Zimmet 1998), glycosylated haemoglobin (an indicator for diabetes) (Seccareccia et al. 2001), triglycerides, cholesterol ratio, and blood pressure (Chobanian et al. 2003) are risk factors of cardiovascular disease (CVD). For each of the single biomarkers, a binary indicator of bad health is created. A score of “1” is assigned to those with “high-risk” values and a score of “0” is assigned to those with “lower risk” values. Values assigning high and low risk are based on cut-off values commonly accepted in clinical practice and the literature for Chinese or Asians (see Table 1). However, each of these biomarkers only measures the potential for a specific type of disease, their effects on general health could be fairly small and a type II error may present. Hence, we also include AL as a summary measure representing the number of biomarkers falling within high-risk values. The AL in this study refers to the group allostatic load index (Juster et al. 2010) which is equal to the sum of “high-risk” conditions weighted by the number of non-missing values. Respondents missing more than three biomarkers are excluded (15%). AL is natural log transformed to reduce skewness, and subsequently standardised for ease of interpretation of results. A number of variables has missing or unknown values, the highest level being reached for household income (5.8%). After deleting these cases we are left with 16,249 respondents for SRH models; the sample size for biomarker models varies from 10,802 to 12,827.

**Table 1:** Cut-off points for high risk values of individual biomarkers

Variable	Cut-off points
Abdominal girth	Waist >80 cm for women, >90 cm for men
Diabetes	Glycosylated haemoglobin $\ge$6.5%, or taking medicine
High blood pressure	Diastolic >90 and systolic >140 mmHg in three measurements, or taking medicine
High triglycerides	$\ge$50 mg/dl
High cholesterol ratio	Total to HDL $\ge$5.92
High C-reactive protein	$\ge$10 mg/L
Weak grip strength	<20 kg women, 30 kg men
Allostatic load	Sum of the number of high risk condition (including poor self-rated health) weighted by number of non-missing observations

Cut-off points used to define “high risk” health conditions

Source: Rosero-Bixby and Dow (2009)

### Provincial-level independent variables

Most provincial-level information is obtained from the 2012 Chinese Statistics Yearbook. Nine variables are selected from available official statistics to reflect the economic situation and healthcare organisation of each province, including GDP per capita, urban/rural median income, level of urbanisation (measured by the proportion of a province’s population living in urban areas), government expenditure for health care, number of hospitals, primary care institutes and doctors weighted by provincial population, and the ratio of government expenditure for health care to total government expenditure. Four variables—GDP per capita, urban median income, rural median income, and level of urbanisation—transformed statistically using natural logarithm. To limit the number of provincial-level variables and to avoid multicollinearity problems, principal component analysis is carried out, with two components extracted, which we interpret as representing a general economic development dimension and a health infrastructure dimension.

Consistent with the literature regarding income inequalities, we use the Gini coefficient. However, given that there are no official published data on Gini coefficients at the provincial level, we constructed Gini coefficients using CHARLS. More specifically, we use equivalised household income using the square root scale and calculate the Gini coefficients for each of the 28 provinces using the package INEQDECO in Stata 12.0, taking the design-weights into account. The Gini coefficient for the *j*th province is computed as (Jenkins 2015):

1 $${G_j}=1+\frac{1}{{{n_j}}} - \left( {\frac{2}{{n_j^2 \cdot {\mu _j}}}} \right)\mathop \sum \limits_{i=1}^{{n_j}} \left( {{n_j}+1 - i} \right){y_{ij~}},\quad j=1,2, \ldots ,k~~$$

In (1) *n*_*j*_ represents the number of households in the province, ${\mu _j}$ is the average equivalised household income, and *y*_*ij*_ denotes the equivalised household income for household *i* in province *j* (with households sorted by their income).

### Individual-level control variables

At the individual level, we use three SES-related control variables: urban/rural residency, education, and equivalized household income. Other socio-demographic covariates include: age, age squared, gender, marital status, and living arrangement.

### Analytical strategy

We use a set of two-level logit regression models using the same specification for different outcomes variables, with individuals nested in provinces. The generic equation for a binary health outcome variable *y*_*ij*_ for the individual *i* living in province *j* has the following form:

2 $$\log \frac{{\Pr \left( {{y_{ij}}=1} \right)}}{{1 - \Pr \left( {{y_{ij}}=1} \right)}}={\gamma _{01}}{X_{ij}}+{\gamma _{02}}{W_j}+{\mu _{0j}}$$

where *X*_*ij*_ is a vector of individual-level independent variables; ${W}_{j}$ is a vector of provincial-level variables; ${\gamma }_{01}$ and ${\gamma }_{02}$ are vectors of parameters related, respectively, to individual- and provincial-level covariates; ${\mu _{0j}}$ is a random intercept at the provincial level (with the usual assumptions of normal distribution, independence from observed variables, expected value of zero and variance ${\sigma }_{{\mu }_{0}}^{2}$).

## Results

### Descriptive statistics

**Figure 1:**
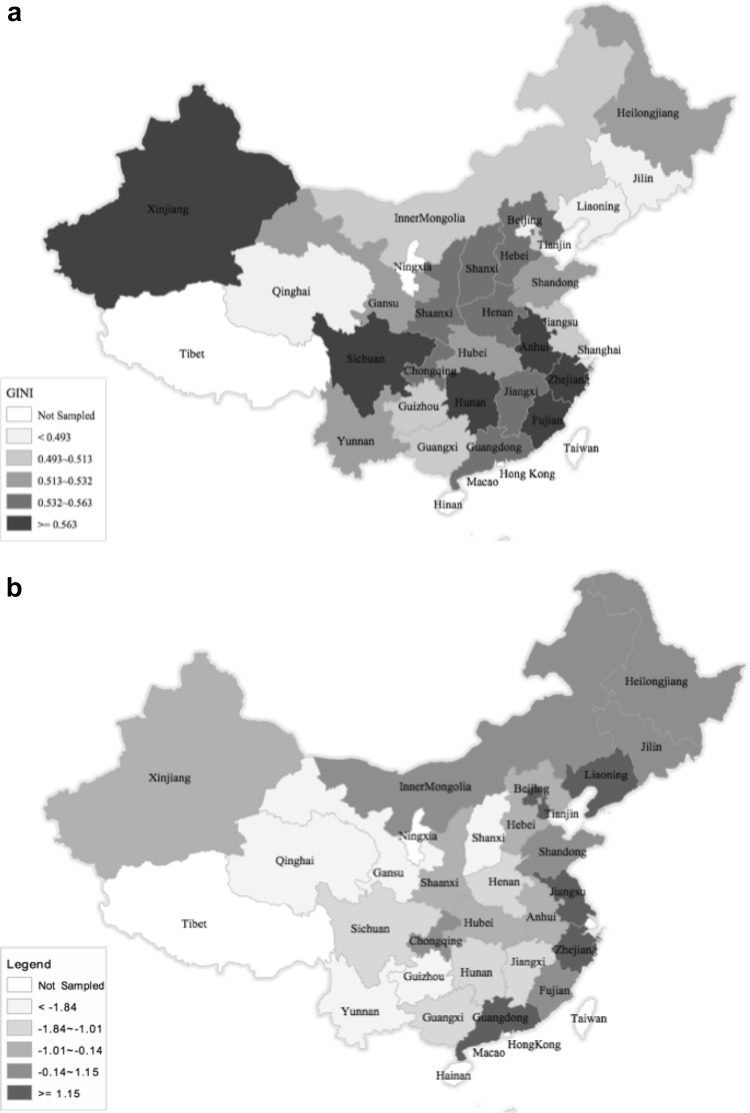

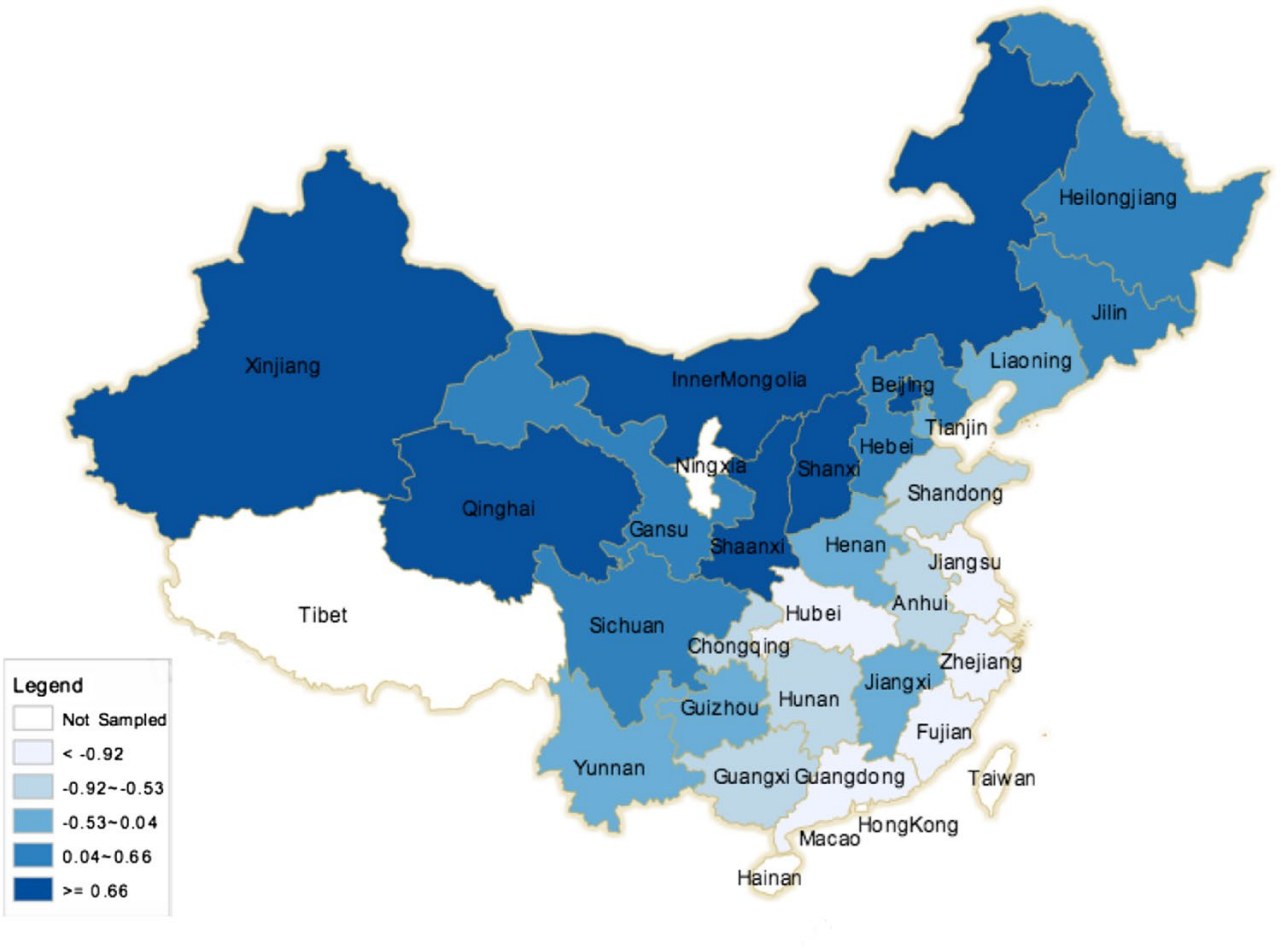

Table 2 shows descriptive statistics for health outcome variables by gender and age. Except for high cholesterol ratio, health outcomes show the expected gender and age gradient. The most prevalent negative outcomes are poor self-rated health, abdominal girth, high triglycerides, weak grip strength, and AL. Weak grip strength in particular has a strong age gradient, and abdominal girth has an exceptionally substantial gender effect. Abdominal girth and high triglycerides have an inverse relationship with age, indicating the possible presence of cohort effects or changes in the composition of the population with ageing in a selection in frailty process (Rosero-Bixby and Dow 2009). Figure 1 maps the values for provincial-level independent variables (for actual values, see Appendix 1). The average Gini coefficient over the 28 provinces in China is 0.54, with Fujian as the most unequal and Shanghai as the most equal (Fig. 1a). These figures are in line with the recent study of Xie and Zhou (2014), who use multiple data sources in 2010 and 2011 and also find that China’s income inequality has reached very high levels with the overall Gini coefficient in the range of 0.53–0.55.

**Table 2:** Descriptive statistics for health outcomes, China Health and Retirement Survey 2011

Health outcome	Sample size	% of negative outcomes	% of negative outcomes by gender			% of negative outcomes by age		
			Male	Female	*χ* ^2^	45–64	65+	*χ* ^2^
Poor self-rated health	16,249	23.8	20.2	27.2	108.8***	20.5	30.5	193.6***
Abdominal girth	12,827	48.0	28.02	66.12	1900***	49.1	45.6	13.87***
Diabetes	10,893	5.4	5.0	5.8	2.9***	5.0	6.2	6.77**
High blood pressure	10,319	13.0	14.0	12.3	7.0**	12.5	14.3	6.5*
High triglycerides	10,808	27.3	24.6	29.6	34.0***	28.8	24.1	26.4***
High cholesterol ratio	10,802	10.0	10.0	10.1	0.1	10.1	9.7	0.6
High C-reactive protein concentrations	10,815	10.3	5.2	3.7	13.6***	3.7	6.0	31.5***
Weak grip strength	12,477	26.1	25.3	26.9	4.1*	16.9	45.0	1100***

		Mean (SD)	Mean by gender (SD)		*T* test	Mean by age (SD)		*T* test
Allostatic load^a^	10,902	0.7 (0.5)	0.6 (0.5)	0.8 (0.5)	20.1***	0.7 (0.5)	0.8 (0.5)	−8.4***

* *p* < 0.05, ** *p* < 0.01, *** *p* < 0.001

^a^Allostatic load reported here is log transformed

The principal components analysis produced two principal components with eigenvalues greater than one, which explain 75% of the total variance. The first principal component (PC_1_), which accounts for 58% of the total variance, is highly and positively correlated with GDP per capita, urban and rural median income, and level of urbanisation (loadings are 0.41, 0.41, 0.39 and 0.39, respectively). We therefore consider PC_1_ as a measure of overall provincial-level economic circumstances and call it “Economic Development” (ED). The second principal component, PC_2,_ is highly and positively correlated with the number of hospitals, government expenditure on health care, and doctors (loadings are 0.64, 0.53, 0.34, respectively), and for this reason we call it “Health-Related Public Infrastructures” (HRPI). Beijing and Shanghai are the most economically developed provinces, whereas Guizhou is the least economically developed province. As one could expect from Chinese economic geography, there is a clear pattern showing that southern-east coastal regions are the most economically developed area, followed by the north and northeast regions. Central and western regions are the least economically developed areas (Fig. 1b).

However, economically well-developed coastal provinces in the south-east—such as Shanghai—lag behind in terms of HRPI. While the less developed middle and west parts have higher scores on HRPI. This finding in line with previous literature, in particular, Li and Wei (2010) and Evandrou et al. (2014) find that the healthcare level of the prosperous coastal provinces is below the national average.

Table 3 provides descriptive statistics on individual-level covariates. The mean age of respondents is 61 years. Approximately 48% of the respondents are male and 88% are married. Average family size is four, and 40% live in urban areas. 27% have never received formal education, 39% have attended or finished primary school, 21% received middle school education, and 13% have finished high school and above. The average equivalized household income in 2011 was about 25,100 yuan (approximately 3860 USD).

**Table 3:** Descriptive statistics for individual-level independent variables, China Health and Retirement Survey 2011

Variables	Mean/proportion	SD	Min	Max
Age	61.01	9.81	45	103
Male	0.48	0.50	0	1
Marital status (married = 1)	0.88	0.33	0	1
Family size	3.65	1.79	1	16
Urban residents	0.40	0.49	0	1
Illiterate	0.27	0.46	0	1
Low education	0.39	0.49	0	1
Intermediate education	0.21	0.41	0	1
High education	0.13	0.33	0	1
Household income (1000 yuan)	25.10	46.46	0	2700
Quintile: 1st (1000 yuan)	1.16	0.87	0	3
Quintile: 2nd (1000 yuan)	6.36	2.20	30.11	30.30
Quintile: 3rd (1000 yuan)	15.12	3.17	10.32	20.91
Quintile: 4th (1000 yuan)	28.68	5.10	20.92	38.40
Quintile: 5th (1000 yuan)	74.47	85.45	38.40	2700

Source: 2011 CHARLS Wave 1 (Baseline)

### Results—self-rated health (SRH)

Our regression models show that ED is negatively associated with poor SRH (Table 4, Model 1). Income inequality is not significantly associated with SRH, even when we include individual-level controls (Models 2, 6). On the contrary, HRPI is positively associated with poor health outcomes (Model 3). The HRPI effect remains significant even after controlling for provincial ED and individual covariates (Models 4, 8). Individual-level covariates show expected and consistent effects, with being older, a woman, and of lower SES associated with poor SRH.

**Table 4:** Two-level logit models for self-reported health (poor health = 1, odds ratios, *N* = 16,249), China Health and Retirement Survey 2011

Estimates	Covariates unadjusted				Covariates adjusted			
	Model 1	Model 2	Model 3	Model 4	Model 5	Model 6	Model 7	Model 8
Provincial-level explanatory variables								
ED	0.91*** (0.02)			0.92** (0.02)	0.95* (0.02)			0.96* (0.02)
Gini coefficient		1.94 (2.06)		1.63 (1.51)		0.95 (0.91)		1.48 (1.29)
HRPI			1.09*** (0.05)	1.10*** (0.05)			1.13**(0.05)	1.14** (0.05)
Individual-level covariates								
Socio-demographic								
Age					1.05*(0.02)	1.05*(0.02)	1.05*(0.02)	1.05*(0.02)
Male					0.71*** (0.03)	0.72*** (0.03)	0.72*** (0.03)	0.72*** (0.03)
Married					1.05 (0.06)	1.04 (0.06)	1.04 (0.06)	1.05 (0.06)
Household size					1.02*(0.01)	1.03* (0.01)	1.03* (0.01)	1.02*(0.01)
Socio-economic status								
Urban resident					0.73***(0.03)	0.72***(0.03)	0.72***(0.03)	0.72***(0.03)
Education (Ref.=Illiterate)								
Low education					0.89* (0.04)	0.89* (0.04)	0.89* (0.04)	0.89* (0.04)
Intermediate education					0.72*** (0.05)	0.71*** (0.05)	0.71*** (0.05)	0.71*** (0.05)
High education					0.53*** (0.04)	0.53*** (0.04)	0.52*** (0.04)	0.53*** (0.04)
Income quintile (Ref. = 1st )								
2nd					0.97 (0.05)	0.97 (0.05)	0.97 (0.05)	0.97 (0.05)
3rd					0.79*** (0.05)	0.79*** (0.05)	0.79*** (0.05)	0.79*** (0.05)
4th					0.65*** (0.04)	0.65*** (0.04)	0.65*** (0.04)	0.65*** (0.04)
5th					0.51***(0.04)	0.51***(0.04)	0.51***(0.04)	0.51***(0.04)
ICC	0.012	0.014	0.014	0.012	0.012	0.012	0.012	0.012
Level 2 variance	0.23***(0.04)	0.28***(0.05)	0.26***(0.05)	0.19*** (0.04)	0.22***(0.04)	0.24***(0.04)	0.20*** (0.04)	0.18***(0.04)
BIC	17697.46	17707.18	17705.15	17713.28	17010.4	17013.88	17006.69	17022.37

Robust standard errors are reported in parentheses

Significance levels: ^†^10%, *5%, **1%, ***0.1%

### Results—biomarkers and allostatic load

Table 5 shows the results of a series of parallel models, each with a different outcome variable and containing the same covariates as Model 8 in Table 4. Compared to the results on SRH, results using biomarkers tend to have a weaker statistical significance, possibly because for the seven single-biomarker models, false negative errors may occur due to an insufficient sample size. At the provincial level, a higher level of income inequality is associated with low risks of hypertension and high triglycerides. Provincial-level ED is positively associated with high risks of abdominal girth and high cholesterol ratio. HRPI is significantly associated with outcomes in 4 out of 8 cases—however, with opposing effects. Higher levels of HRPI are associated with abdominal girth, high blood pressure, high C-reactive protein concentrations and allostatic load.

**Table 5:** Two-level logit models for single biomarkers (poor health = 1, odds ratios), and two-level linear model for allostatic load (Coefficient), China Health and Retirement Suvey 2011

Estimates	Abdominal girth	Diabetes	Hypertension	High triglycerides	High cholesterol ratio	High C-reactive protein concentration	Weak grip strength	Allostatic load
Provincial-level explanatory variables								
Gini coefficient	0.63 (0.94)	0.32 (0.48)	0.03** (0.04)	0.13* (0.14)	1.46 (1.87)	1.82 (2.61)	1.40 (2.13)	−0.66 (0.44)
ED	1.08* (0.04)	1.02 (0.04)	0.96 (0.03)	0.99 (0.03)	1.06* (0.04)	1.05 (0.04)	1.02 (0.04)	0.01 (0.01)
HRPI	1.12* (0.08)	1.05 (0.08)	1.11* (0.06)	1.03 (0.06)	1.02 (0.07)	1.07* (0.05)	1.08(0.05)	0.03*** (0.01)
Individual-level covariates								
Socio-demographic								
Age	1.05*(0.02)	1.28*** (0.07)	1.01* (0.01)	0.99** (0.01)	1.00 (0.01)	1.03***(0.01)	1.08***(0.01)	0.01***(0.01)
Male	0.17*** (0.01)	0.77** (0.07)	1.20** (0.08)	0.77*** (0.04)	0.99*** (0.07)	1.40** (0.14)	0.94 (0.05)	−0.34*** (0.02)
Married	1.22** (0.08)	1.41* (0.21)	0.74** (0.07)	1.06 (0.08)	1.18 (0.13)	1.09 (0.16)	0.77*** (0.05)	−0.01 (0.03)
Household size	0.97** (0.01)	0.98 (0.03)	1.00 (0.02)	1.00 (0.01)	1.05* (0.02)	0.99 (0.83)	1.03* (0.01)	−0.01 (0.01)
Socio-economic status								
Urban resident	1.72*** (0.08)	1.74*** (1.06)	1.19** (0.08)	1.32*** (0.06)	1.46***(0.10)	1.07 (0.11)	0.93 (0.05)	0.15***(0.02)
Education (Ref.=Illiterate)								
Low education	1.10^*†*^ (0.06)	0.97 (0.11)	0.98 (0.08)	0.98 (0.06)	1.12 (0.10)	0.88 (0.10)	0.77*** (0.04)	−0.01 (0.02)
Intermediate education	1.21** (0.08)	1.27^*†*^ (0.18)	0.97 (0.10)	1.04 (0.08)	1.13 (0.12)	0.89 (0.14)	0.61*** (0.05)	−0.02 (0.03)
High education	1.17* (0.10)	1.14 (0.19)	0.88 (0.11)	0.97 (0.09)	1.17 (0.15)	0.62* (0.13)	0.56*** (0.06)	−0.07* (0.03)
Income quintile (Ref. = 1st )								
2nd	0.98 (0.06)	0.93 (0.13)	0.89 (0.08)	0.96 (0.07)	0.94 (0.10)	0.85 (0.12)	0.77*** (0.05)	−0.05^*†*^ (0.03)
3rd	1.01 (0.07)	0.99 (0.14)	0.93 (0.09)	0.95 (0.07)	1.03^*†*^ (0.07)	0.73* (0.11)	0.69*** (0.05)	−0.07* (0.03)
4th	1.10 (0.07)	0.14 (0.16)	0.81* (0.08)	1.01 (0.07)	1.09* (0.04)	0.83 (0.13)	0.62*** (0.05)	−0.05^*†*^ (0.03)
5th	1.15 (0.08)	1.09 (0.16)	0.88 (0.09)	1.13 (0.09)	1.10 (0.10)	0.85 (0.14)	0.53***(0.04)	−0.05 (0.03)
Intercept								−0.25* (0.19)
ICC	0.017	0.02	0.015	0.012	0.017	0.019	0.019	0.005
Sample size	12,827	10,893	10,319	10,808	10,802	10,815	12,477	10,902

Robust standard errors are reported in parentheses

Significance levels: ^†^10%, *5%, ** 1%, ***0.1%

Individual-level SES is not statistically significantly associated with all biomarkers, in contrast to its consistent effect on self-rated health and allostatic load. SES is associated with grip strength, with higher educational attainment and income level associated with stronger grip strength. For other biomarkers, higher income only matters for lower risk of high blood pressure in the low and highest quintile income group. Education also loses its significance in predicting most of these outcomes. In three of the eight of the health indicators in the table, there is no significant joint SES effect (*p* > 0.05). For the others, the SES gradient sometimes behaves as expected with below-one odds ratio for the high-SES categories and above one for the low SES. We observe a reversal for abdominal girth, with higher level of income linked to higher risk.

## Discussion

This study uses multi-level data to investigate the association between economic development (ED), income inequality, and health-related public infrastructure (HRPI), and various health outcomes for Chinese midlife and older adults. Consistently with previous research, the three contextual factors vary greatly between provinces (Evandrou et al. 2014). Coastal areas are more economically developed than inland areas, but the level of HRPI of these wealthy areas is below the national average. Income inequality has reached very high levels in China, and is negatively associated with ED (corr = −0.41; *p* < 0.01), indicating that the large increase in economic inequality may constrain the potential for rapid economic growth (Xie and Zhou 2014).

We show that a higher level of provincial ED is positively correlated with SRH, but is not significantly associated with most of the biomarker outcomes. ED is positively associated with high risks of abdominal girth and high cholesterol ratio. The results are consistent with research in less developed countries, i.e. higher average income can be a marker of risk for obesity and obesity-induced heart diseases (Sayeed et al. 1997). Individual-level covariates further confirm that abdominal girth and high cholesterol ratio are more prevalent among urban residents with higher SES. Dietary pattern and physical activity behaviours are likely to be relevant explanations. People from wealthier provinces tend to consume unhealthier food, and exercise less. People from less developed province, and with lowest SES tend to reside in the rural area, consuming less total food, less fat and oil, and more vegetables (Xu et al. 2006). Rural residents also tend to actively engage in agricultural activities, thus are less likely to be overweight or obese.

Consistent with recent research on less developed countries (Biggs et al. 2010; Rajan et al. 2013), we find no supportive evidence in favour of the income inequality hypothesis. In fact, regional income distribution is not correlated with health conditions for most of our health outcomes, and the only statistically significant relationship runs in a direction that is in contradiction to the income inequality hypothesis. More specifically, we find that greater inequality is correlated with better health outcomes in blood pressure and triglycerides. This positive association between income inequality and health may reflect the impact of income inequality on economic growth. Apart from observing a moderate negative relationship between income inequality and ED, economists have also argued that income inequality affects the economy negatively, by depriving the poor of the ability to accumulate human capital, generating political and economic instability that reduces investment, and impedes the social consensus required to adjust to shocks and sustain growth (Ostry et al. 2014). Provinces with relatively equal income distribution, thus tend to have higher level of economic development, and as we explained before, may have a negative impact on diseases related to over-nutrition.

Our result seems to corroborate a materialist pathway, i.e. that societal economic level of development is more important than inequality in determining individual health status (Preston 1975). However, it would be premature to conclude that income inequality does not matter for the health of midlife and older adults in China. Pickett and Wilkinson (2015) suggest that income inequality may not be an independent determinant of health, but it might strengthen the many known and unknown causal process through which social class imprints it self on people throughout life. Moreover, Subramanian and Kawachi (2004) provide evidence that income inequality may have a lagged effect on health, possibly of up to 15 years. Our study thus suffers from the time-frame limitation that the effects of income inequality on health may operate over many years.

Throughout our models, higher levels of health-related public infrastructures are robustly associated with poor health. The association indicates the likely presence of reversed causality. The Chinese government’s efforts to increase investment as well as effectiveness in areas with lower level of public health (Zhu 2013). Another explanation is that the current settings of health infrastructure may not be efficient in treating the chronic diseases measured in our study. We recognise that future research is required to illuminate this puzzling relations.

Our results are suggestive, albeit not conclusive, regarding the effects of aggregate-level socioe-conomic factors on subjective health outcomes and biomarkers. It is indeed likely that the causal mechanisms and pathways linking aggregate-level socio-economic factors to each biomarker are different. Additional research is needed to generate theories and evidence on the specific mechanisms.

To summarise, this study helps to extend our knowledge of how provincial variations in ED, income inequality, and HRPI, on top of individual characteristics, can affect individual health status according to a range of health indicators. These findings point to significant policy implications. First, interventions to alleviate the effects of poverty on health in China are likely to be of greatest benefit if targeted at the poorest regions. Second, we speculate that policy makers should also focus on dietary and life style issues, as the availability of unhealthy food and sedentary behaviours may have become a by-product of economic growth. While inequality cannot predict most of the health outcomes, it is moderately negatively connected to economic growth, and thus may undermine the effect of economy on health. Economic policies narrowly focused on growth, are likely to be insufficient in improving Chinese midlife and older adults’ health.

## Electronic supplementary material

Below is the link to the electronic supplementary material.

Supplementary material 1 (DOCM 887 KB)
