# Supplementary material for: Health of midlife and older adults in China: the role of regional economic development, inequality, and institutional setting
Source: Int J Public Health. 2017 Apr 22;62(8):857–67. doi: 10.1007/s00038-017-0970-9 (PMC5641278; doi:10.1007/s00038-017-0970-9)
Supplement: Supplementary file 1 — Supplementary material 1 (DOCM 887 KB) [file 38_2017_970_MOESM1_ESM.docm]

**Health of midlife and older adults in China: the role of regional economic development, inequality, and institutional setting**

**Xuejie Ding^1^** , **Francesco C. Billari^2^ and Stuart Gietel-Basten^3^**

**^1^ Department of Sociology, University of Oxford; ^2^ Carlo F. Dondena Centre for Research on Social Dynamics and Public Policies and Department of Policy Analysis and Public Management, Bocconi University; ^3^ Department of Social Policy and Intervention, University of Oxford**

***Abstract***

*Objectives*

*To document the association between economic development, income inequality, and health-related public infrastructure, and health outcomes among Chinese adults in midlife and older age.*

*Methods*

*We use a series of multi-level regression models with individual-level baseline data from the China Health and Retirement Longitudinal Survey (CHARLS). Provincial-level data are obtained both from official statistics and from CHARLS itself. Multi-level models are estimated with different subjective and objective health outcomes.*

*Results*

*Economic growth is associated with better self-rated health, but also with obesity. Better health infrastructure tends to be negatively associated with health outcomes, indicating the likely presence of reverse causality. No supportive evidence is found for the hypothesis that income inequality leads to worse health outcomes.*

*Conclusions*

*Our study shows that on top of individual characteristics, provincial variations in economic development, income inequality, and health infrastructure are associated with a range of health outcomes for Chinese midlife and older adults. Economic development in China might also bring adverse health outcomes for this age group; as such specific policy responses need to be developed.*

*Keywords:*

*Biomarkers; China; Economic development; Health-related public infrastructure; Income inequality; Midlife and older adults*

**Introduction**

Three overarching ideas have been proposed to understand health inequality across time and place. First, a higher level of economic development is seen as leading to better health outcomes (Preston 1975). Second, in a thesis that has been widely discussed, greater income inequality is seen to worsen health outcomes, through both psychosocial and material mechanisms (Wilkinson and Pickett 2010). Third, the provision of health services and infrastructure is considered a key determinant of health (Anand and Ravallion 1993), which may even moderate the effect of economic development and income inequality.

It is widely acknowledged that economic development allows to improve the quality of goods and services such as food, health care, medical services, etc., which in turn leads to improved health and nutrition among the population (Anand and Ravallion 1993). However, recent research shows that economic growth might also foster the prevalence of chronic diseases, such as obesity, diabetes and their complications. Evidence shows that the urbanisation and economic progress in China have led to a radical reduction in overall and occupational physical activity (Ng, Norton and Popkin 2009; Sherif and Sumpio 2015), as well as an increase in fat consumption.

The link between income inequality and health is also controversial, both at the theoretical level and empirically. Wilkinson and Pickett (2010) suggest that individuals who live in a more equal society have on average better health outcomes than their counterparts living in less equal societies. This idea, formulated with specific reference to developed societies, is hypothesised to work through both psychosocial and material mechanisms. More specifically, income inequality would influence health through relative deprivation. For individuals with relatively low incomes, inequality generates negative emotions such as shame and stress that harm health through “psycho-neuro-endocrine” mechanisms, and induces unhealthy behaviours such as smoking and alcohol abuse. Relative deprivation also impairs health for the whole population, through a reduction of social capital and social cohesion: in more unequal societies, participation in community activities is reduced, and trust and efficacy within a community are weakened, with potential effects on the health of the entire population, including those higher up the income range (Kawachi and Kennedy 1999). The evidence from the large body of empirical work testing the income inequality hypothesis is however mixed. While Pickett and Wilkinson (2015) insist on the relationship between inequality and population health being causal, methodological concerns and empirical evidence have cast doubts on the causal nature of this relationship. Studies using large and high-quality data sets found that the effects of income inequality, which are significant in OLS regression models, tend to disappear or become markedly weaker when more appropriate fixed-effect or multi-level regression models are used (Beckfield 2004). Mellor and Milyo (2003), in a study on the US, added a variety of controls to their analysis and found that income inequality has no significant effect on health. Biggs et al. (2010) and Rajan et al. (2013) revisited the income inequality hypothesis in Latin America and India and found no evidence supporting the claim that income inequality is detrimental to health in these less developed global settings.

The provision of health-related public infrastructure [HRPI] has been shown to make a difference to health outcomes through its link to economic development and income inequality. Higher levels of economic development allow greater public investments in HRPI at the aggregate-level, and make out-of-pocket expenditures in medicines and services at the individual-level more affordable (Dollar and Kraay 2002). Societies with higher levels of income inequality, with the US as a textbook case, tend to spend less on improving public health or human capital development. This lower level of public investment weakens the opportunities that individuals have to improve their living standards and health (Kentikelenis, Stubbs, and King 2015). Improved health services and infrastructures make healthcare more affordable and accessible, with the potential to buffer the negative effects arising from income inequality (Ellwardt et al. 2014).

In summary, the literature emphasizes three interdependent aggregate-level determinants of health: economic development, income inequality and investment in health infrastructure. Although it has been widely recognized that individual characteristics explain most of the variation in health disparity, this study aims to understand the effects of these contextual factors for a specific group: Chinese midlife and older adults (age 45 and above). In China, the rapidly growing elderly population poses great challenges for global health. Over the past few decades, the country has experienced unprecedented economic growth, accompanied by dramatic increase in income inequality (Li and Zhu 2006). Although a high rate of improvement in HRPI has occurred in China since the 1990s (Liu 2004), the ever-increasing share of personal health and medical expenditure still poses a challenge for the government (Zhang and Kanbur, 2005). It is hence paramount to examine whether the effects of economic development on health are modified by inequality or health infrastructure.

We specifically focus on midlife and older adults. Midlife is a period in life in which limited physical functioning and manifest chronic diseases start to become widespread. Furthermore, this period of life is linked to growing healthcare needs, which increase burdens on existing formal and informal health infrastructures (Muramatsu 2003). Midlife and older adults are therefore vulnerable to socio-economic disadvantages and changes in their living environment, and are particularly relevant to understanding the relationship between economic growth, inequality and medical infrastructure on health (Mosquera et al. 2016).

Previous studies of the link between wealth/inequality and health link have relied on survey data, or hospital records. While our study also uses self-rated health (SRH), we additionally employ biomarker data to measure health risks. The utilization of a series of biomarkers provides some attractive features. Biomarkers are measured objectively, minimizing systematic reporting errors caused by bias and preferences. They provide an identification of pre-disease pathways, since physiological processes are often below the individual’s threshold of perception. We also include allostatic load (AL) – a comprehensive index that incorporates multiple biomarker risk factors, which is expected to better predict future health risks than any single risk factor by itself. However, the mechanisms underlying the links between income inequality and specific biomarkers are not yet clear. Any (or all) of the aforementioned mechanisms could mediate the possible relation between income inequality and biomarkers in cardiovascular diseases and physical functions. “Psycho-neuro-endocrine” factors could potentially affect diet choice and rates of physical activity, consequently affecting blood pressure, inflammation and grip strength. In addition, less egalitarian provinces may invest less resources in promoting and maintaining healthy behaviours, and may also invest less in health education and preventive care. The exposure to economic underdevelopment and income inequality accumulates throughout the life course, and places demands on the biological system (e.g., cardiovascular and metabolic system), ultimately leading to greater system dysregulation, subsequently enhancing risk for poor health and functioning (Gruenewald et al., 2012). The prevention, diagnosis, and long-term treatment of non-communicable and chronic diseases, as well as physical functioning indicated by biomarkers, are heavily relying on health infrastructures (Blankart, 2012). Some recent studies focusing on the United States found that risk factors for cardiovascular diseases, including hypertension, diabetes, and grip strength, show variation at regional levels and are associated with state-level income inequality (Cushman et al., 2009; Diez-Roux, Link and Northridge., 2000; De Vries, Blane and Netuveli., 2014). To our knowledge, this paper is the first to study the associations between income inequality, economic development, health infrastructure and health as measured through biomarkers among Chinese midlife and older adults.

**Methods**

***Data***

Given our multi-level framework, we need data both at the individual and aggregate (provincial) level. Individual-level SRH and physical measures of girth, blood pressure, handgrip strength, and covariates comes from the 2011 national baseline survey of the Chinese Health and Retirement Longitudinal Study (CHARLS). CHARLS, modelled after the US Health and Retirement Study, provides a wide range of information, from socio-economic status and social support to health conditions for a randomly-selected and nationally-representative sample of Chinese residents age 45 and above, living in households. The total analytical sample of the baseline national wave comprises 17,368 individuals across 28 provinces. Among the interviewed subjects, 11,635 individuals (67%) accepted to provide a blood sample, and therefore allow us to have biomarker information on glycosylated haemoglobin, triglycerides, cholesterol ratio and C-reactive protein. Since women had a slight higher blood response rate than men (69% versus 65%), we use the sample weights that the CHARLS team has calculated to correct for household and individual non-response as well as non-participation in the blood collection (for details, see Zhao et al. 2014).

*Outcome variables*

We study SRH, seven single biomarkers, and AL as outcomes. SRH and single biomarkers are coded as dichotomous indicators. AL is treated as continuous variable. The seven single biomarkers are: girth, glycosylated hemoglobin, blood pressure, triglycerides, cholesterol ratio, C-reactive protein, and grip strength. These biomarkers are good predictors of diseases. For instance, Ridker (2003) found that C-reactive protein helps predict risk of heart attack and stroke. Abdominal girth (Alberti and Zimmet 1998), glycosylated hemoglobin (an indicator for diabetes) (Seccareccia et al. 2001), triglycerides, cholesterol ratio, and blood pressure (Chobanian et al. 2003) are risk factors of cardiovascular disease (CVD). For each of the single biomarkers, a binary indicator of bad health is created. A score of “1” is assigned to those with “high-risk” values and a score of “0” is assigned to those with “lower risk” values. Values assigning high and low risk are based on cut-off values commonly accepted in clinical practice and the literature for Chinese or Asians (see Table 1). However, each of these biomarkers only measures the potential for a specific type of disease, their effects on general health could be fairly small and a type II error may present. Hence we also include AL as a summary measure representing the number of biomarkers falling within high-risk values. The AL in this study refers to the group allostatic load index (Juster, McEwen, and Lupien 2010), which is equal to the sum of “high-risk” conditions weighted by the number of non-missing values. Respondents missing more than three biomarkers are excluded (15%). AL is natural-log transformed to reduce skewness, and subsequently standardised for ease of interpretation of results. A number of variables has missing or unknown values, the highest level being reached for household income (5.8%). After deleting these cases we are left with 16,249 respondents for SRH models; the sample size for biomarker models varies from 10,802 to 12,827^[[1]](#footnote-1)^.

*Provincial-Level Independent Variables*

Most provincial-level information is obtained from the 2012 Chinese Statistics Yearbook. Nine variables are selected from available official statistics to reflect the economic situation and healthcare organisation of each province, including GDP per capita, urban/rural median income, level of urbanisation (measured by the proportion of a province’s population living in urban areas), government expenditure for health care, number of hospitals, primary care institutes and doctors weighted by provincial population, and the ratio of government expenditure for health care to total government expenditure. Four variables – GDP per capita, urban median income, rural median income, and level of urbanisation - transformed statistically using natural logarithm. In order to limit the number of provincial-level variables and to avoid multicollinearity problems, Principal Component Analysis is carried out, with two components extracted, which we interpret as representing a general economic development dimension and a health infrastructure dimension.

Consistent with the literature regarding income inequalities, we use the Gini coefficient. However, given that there are no official published data on Gini coefficients at the provincial level, we constructed Gini coefficients using CHARLS. More specifically, we use equivalised household income using the square root scale and calculate the Gini coefficients for each of the 28 provinces using the package INEQDECO in Stata 12.0, taking the design-weights into account. The Gini coefficient for the *j*-th province is computed as (Jenkins 2010):

$$G_{j}=1+\frac{1}{n_{j}}-\left( \frac{2}{n_{j}^{2}\cdot\mu_{j}} \right)\sum_{i=1}^{n_{j}} \left( n_{j}+1-i \right)y_{ij}, j=1,2,.,\cdot\cdot\cdot,k (1)$$

In (1) $n_{j}$represents the number of households in the province,$\mu_{j}$ is the average equivalised household income, and $y_{ij}$denotes the equivalised household income for household *i* in province *j* (with households sorted by their income).

*Individual-level control variables*

At the individual level, we use three SES-related control variables: urban/rural residency, education, and equivalized household income. Other socio-demographic covariates include: age, age squared, gender, marital status, and living arrangement.

*Analytical Strategy*

We use a set of two-level logit regression models using the same specification for different outcomes variables, with individuals nested in provinces. The generic equation for a binary health outcome variable $y_{ij}$for the individual *i* living in province *j* has the following form:

$log\frac{Pr\left( y_{ij}=1 \right)}{1-Pr\left( y_{ij}=1 \right)}=\gamma_{01}X_{ij}+\gamma_{02}W_{j}+\mu_{0j}$（2）

where: $X_{ij}$is a vector of individual-level independent variables; $W_{j}$ is a vector of provincial-level variables; $\gamma_{01}$and $\gamma_{02}$ are vectors of parameters related respectively to individual- and provincial-level covariates; $\mu_{0j}$is a random intercept at the provincial level (with the usual assumptions of normal distribution, independence from observed variables, expected value of zero and variance $\sigma_{\mu_{0}}^{2}$).

**Results**

*Descriptive statistics*

Table 2 shows descriptive statistics for health outcome variables by gender and age. Except for high cholesterol ratio, health outcomes show the expected gender and age gradient. The most prevalent negative outcomes are poor self-rated health, abdominal girth, high triglycerides, weak grip strength, and AL. Weak grip strength in particular has a strong age gradient, and abdominal girth has an exceptionally substantial gender effect. Abdominal girth and high triglycerides have an inverse relationship with age, indicating the possible presence of cohort effects or changes in the composition of the population with aging in a selection in frailty process (Rosero-Bixby and Dow 2009). Figure 2 maps the values for provincial-level independent variables (for actual values, see *Appendix 1*). The average Gini coefficient over the 28 provinces in China is 0.54, with Fujian as the most unequal and Shanghai as the most equal (Figure 2a). These figures are in line with the recent study of Xie and Zhou (2014), who use multiple data sources in 2010 and 2011 and also find that China’s income inequality has reached very high levels with the overall Gini coefficient in the range of 0.53- 0.55.

The Principal Components Analysis produced two principal components with eigenvalues greater than one, which explain 75% of the total variance. The first principal component (PC_1_), which accounts for 58% of the total variance, is highly and positively correlated with GDP per capita, urban and rural median income, and level of urbanisation (loadings are 0.41, 0.41, 0.39 and 0.39, respectively). We therefore consider PC_1_ as a measure of overall provincial-level economic circumstances and call it “Economic Development” (ED). The second principal component, PC_2,_ is highly and positively correlated with the number of hospitals, government expenditure on health care, and doctors (loadings are 0.64, 0.53, 0.34, respectively), and for this reason we call it “Health-Related Public Infrastructures” (HRPI). Beijing and Shanghai are the most economically developed provinces, whereas Guizhou is the least economically developed province. As one could expect from Chinese economic geography, there is a clear pattern showing that southern-east coastal regions are the most economically developed area, followed by the north and northeast regions. Central and western regions are the least economically developed areas (Figure 2b).

However, economically well-developed coastal provinces in the south-east – such as Shanghai – lag behind in terms of HRPI. While the less developed middle and west parts have higher scores on HRPI. This finding in line with previous literature, in particular, Li and Wei (2010) and Evandrou et al. (2014) find that the healthcare level of the prosperous coastal provinces is below the national average.

Table 3 provides descriptive statistics on individual-level covariates. The mean age of respondents is 61 years. Approximately 48% of the respondents are male and 88% are married. Average family size is four, and 40% live in urban areas. 27% have never received formal education, 39% have attended or finished primary school, 21% received middle school education, and 13% have finished high school and above. The average equivalized household income in 2011 was about 25,100 yuan (approximately 3,860 USD).

*Results – self-rated health (SRH)*

Our regression models show that ED is negatively associated with poor SRH (Table 4, Model 1). Income inequality is not significantly associated with SRH, even when we include individual-level controls (Models 2, 6). On the contrary, HRPI is positively associated with poor health outcomes (Model 3). The HRPI effect remains significant even after controlling for provincial ED and individual covariates (Models 4, 8). Individual-level covariates show expected and consistent effects, with being older, a woman, and of lower SES associated with poor SRH.

*Results – biomarkers and allostatic load*

Table 5 shows the results of a series of parallel models, each with a different outcome variable and containing the same covariates as Model 8 in Table 4. Compared to the results on SRH, results using biomarkers tend to have a weaker statistical significance, possibly because for the seven single-biomarker models, false negative errors may occur due to an insufficient sample size. At the provincial level, a higher level of income inequality is associated with low risks of hypertension and high triglycerides. Provincial-level ED is positively associated with high risks of abdominal girth and high cholesterol ratio. HRPI is significantly associated with outcomes in 4 out of 8 cases—however with opposing effects. Higher levels of HRPI are associated with abdominal girth, high blood pressure, high C-reactive protein concentrations and allostatic load.

Individual-level SES is not statistically significantly associated with all biomarkers, in contrast to its consistent effect on self-rated health and allostatic load. SES is associated with grip strength, with higher educational attainment and income level associated with stronger grip strength. For other biomarkers, higher income only matters for lower risk of high blood pressure in the low and highest quintile income group. Education also loses its significance in predicting most of these outcomes. In three of the eight of the health indicators in the table, there is no significant joint SES effect (p > .05). For the others, the SES gradient sometimes behaves as expected with below-one odds ratio for the high-SES categories and above one for the low SES. We observe a reversal for abdominal girth, with higher level of income linked to higher risk.

**Discussion**

This study uses multi-level data to investigate the association between economic development (ED), income inequality, and health-related public infrastructure (HRPI), and various health outcomes for Chinese midlife and older adults. Consistently with previous research, the three contextual factors vary greatly between provinces (Evandrou et al. 2014). Coastal areas are more economically developed than inland areas, but the level of HRPI of these wealthy areas is below the national average. Income inequality has reached very high levels in China, and is negatively associated with ED (corr = -0.41; p < 0.01), indicating that the large increase in economic inequality may constrain the potential for rapid econoimc growth (Xie and Zhou 2014).

We show that a higher level of provincial ED is positively correlated with SRH, but is not significantly associated with most of the biomarker outcomes. ED is positively associated with high risks of abdominal girth and high cholesterol ratio. The results are consistent with research in less developed countries, i.e. higher average income can be a marker of risk for obesity and obesity-induced heart diseases (Sayeed et al. 1997). Individual-level covariates further confirm that abdominal girth and high cholesterol ratio are more prevalent among urban residents with higher SES. Dietary pattern and physical activity behaviours are likely to be relevant explanations. People from wealthier provinces tend to consume unhealthier food, and exercise less. People from less developed province, and with lowest SES tend to reside in the rural area, consuming less total food, less fat and oil, and more vegetables (Xu et al. 2006). Rural residents also tend to actively engage in agricultural activities, thus are less likely to be overweight or obese.

Consistent with recent research on less developed countries (Biggs et al. 2010; Rajan et al. 2013), we find no supportive evidence in favour the income inequality hypothesis. In fact, regional income distribution is not correlated with health conditions for most of our health outcomes, and the only statistically significant relationship runs in a direction that is in contradiction to the income inequality hypothesis. More specifically, we find that greater inequality is correlated with better health outcomes in blood pressure and triglycerides. This positive association between income inequality and health may reflect the impact of income inequality on economic growth. Apart from observing a moderate negative relationship between income inequality and ED, economists have also argued that income inequality affects the economy negatively, by depriving the poor of the ability to accumulate human capital, generating political and economic instability that reduces investment, and impedes the social consensus required to adjust to shocks and sustain growth (Ostry, Berg, and Tsangarides 2014). Provinces with relatively equal income distribution, thus tend to have higher level of economic development, and as we explained before, may have a negative impact on diseases related to over-nutrition.

Our result seems to corroborate a materialist pathway, i.e. that societal economic level of development is more important than inequality in determining individual health status (Preston, 1975). However, it would be premature to conclude that income inequality does not matter for the health of midlife and older adults in China. Pickett and Wilkinson (2015) suggest that income inequality may not be an independent determinant of health, but it might strengthen the many known and unknown causal process through which social class imprints it self on people throughout life. Moreover, Subramanian and Kawachi (2004) provide evidence that income inequality may have a lagged effect on health, possibly of up to 15 years. Our study thus suffers from the time-frame limitation that the effects of income inequality on health may operate over many years.

Throughout our models, higher levels of health-related public infrastructures are robustly associated with poor health. The association indicates the likely presence of reversed causality. The Chinese government’s efforts to increase investment as well as effectiveness in areas with lower level of public health (Zhu 2013). Another explanation is that the current settings of health infrastructure may not be efficient in treating the chronic diseases measured in our study. We recognise that future research is required to illuminate this puzzling relations.

Our results are suggestive, albeit not conclusive, regarding the effects of aggregate-level socioeconomic factors on subjective health outcomes and biomarkers. It is indeed likely that the causal mechanisms and pathways linking aggregate-level socioeconomic factors to each biomarker are different. Additional research is needed to generate theories and evidence on the specific mechanisms.

To summarise, this study helps to extend our knowledge of how provincial variations in ED, income inequality, and HRPI, on top of individual characteristics, can affect individual health status according to a range of health indicators. These findings point to significant policy implications. First, interventions to alleviate the effects of poverty on health in China are likely to be of greatest benefit if targeted at the poorest regions. Second, we speculate that policy makers should also focus on dietary and life style issues, as the availability of unhealthy food and sedentary behaviours may have become a by-product of economic growth. While inequality cannot predict most of the health outcomes, it is moderately negatively connected to economic growth, and thus may undermine the effect of economy on health. Economic policies narrowly focused on growth, are likely to be insufficient in improving Chinese midlife and older adults’ health.

**Acknowledgements**

The authors would like to thank Nicola Barban, Rob Gruijters, Wential Lu, Maria-Giovanna Merli, Melinda Mills, Patrick Präg, Matthias Qian, and two anonymous referees for their comments and suggestions. F.C. Billari was affiliated with the Department of Sociology and Nuffield College, University of Oxford, while conducting this research.

**References**

Alberti KGMM, Zimmet PF (1998) Definition, diagnosis and classification of diabetes mellitus and its complications. Part 1: diagnosis and classification of diabetes mellitus. Provisional report of a WHO consultation. Diabetic Medicine 15(7): 539-553

Anand S, Ravallion M (1993) Human development in poor countries: On the role of private incomes and public services. The Journal of Economic Perspectives 7(1): 133-150

Beckfield J (2004) Does income inequality harm health? New cross-national evidence. Journal of Health and Social Behavior 45(3): 231-248

Biggs B, King L, Basu S, Stuckler D (2010) Is wealthier always healthier? The impact of national income level, inequality, and poverty on public health in Latin America. Social Science and Medicine 71(2): 266-273

Blankart CR (2012) Does healthcare infrastructure have an impact on delay in diagnosis and survival?. Health Policy 105(2): 128-137

Chobanian AV, Bakris GL, Black R et al (2003) Seventh report of the joint national committee on prevention, detection, evaluation, and treatment of high blood pressure. Hypertension 42(6): 1206-1252

Cushman M, McClure LA, Howard VJ et al (2009) Implications of increased C-reactive protein for cardiovascular risk stratification in black and white men and women in the US. Clinical Chemistry 55(9): 1627-1636

De Vries R, Blane D, Netuveli G (2014) Long-term exposure to income inequality: Implications for physical functioning at older ages. European Journal of Ageing 11(1): 19-29

Diez-Roux AV, Link BG, Northridge ME (2000) A multilevel analysis of income inequality and cardiovascular disease risk factors. Social Science and Medicine 50(5): 673-687

Dollar D, Kraay A (2002) Growth is good for the poor. Journal of Economic Growth 7(3): 195-225

Ellwardt L, Peter S, Präg P, Steverink N (2014) Social contacts of older people in 27 European countries: the role of welfare spending and economic inequality. European Sociological Review 30(4): 413-430

Evandrou M, Falkingham J, Feng Z Vlachantoni A (2014) Individual and province inequalities in health among older people in China: Evidence and policy implications. Health and place 30: 134-144

Gruenewald TL, Karlamangla AS, Hu P, Stein-Merkin S, Crandall C, Koretz B, Seeman TE (2012) History of socioeconomic disadvantage and allostatic load in later life. Social Science and Medicine 74(1): 75-83

Jenkins SP (2015) INEQDECO: Stata module to calculate inequality indices with decomposition by subgroup. Statistical Software Components

Juster RP, McEwen BS, Lupien SJ (2010) Allostatic load biomarkers of chronic stress and impact on health and cognition. Neuroscience and Biobehavioral Reviews 35(1): 2-16

Kawachi I, Kennedy BP (1999) Income inequality and health: pathways and mechanisms. Health Services Research 34(1 Pt 2): 215

Kentikelenis AE, Stubbs TH, King LP (2015) Structural adjustment and public spending on health: Evidence from IMF programs in low-income countries. Social Science and Medicine 126: 169-176

Li Y, Wei YD (2010) A spatial-temporal analysis of health care and mortality inequalities in China. Eurasian Geography and Economics 51(6): 767-787

Li H, Zhu Y (2006) Income, income inequality, and health: Evidence from China. Journal of Comparative Economics 34(4): 668-693

Liu, Y (2004) China's public health-care system: facing the challenges. Bulletin of the World Health Organization 82(7): 532-538

Mellor JM, Milyo J (2003) Is exposure to income inequality a public health concern? Lagged effects of income inequality on individual and population health. Health Services Research 38(1p1): 137-151

Mosquera PA, San Sebastian M, Waenerlund AK, Ivarsson A, Weinehall L, Gustafsson PE (2016) Income-related inequalities in cardiovascular disease from mid-life to old age in a Northern Swedish cohort: A decomposition analysis. Social Science and Medicine 149: 135-144

Muramatsu N (2003) County‐level income inequality and depression among older Americans. Health Services Research 38(6p2): 1863-1884

Ng SW, Norton EC, Popkin BM (2009) Why have physical activity levels declined among Chinese adults? Findings from the 1991–2006 China Health and Nutrition Surveys. Social Science and Medicine 68(7): 1305-1314

Ostry MJD, Berg MA, Tsangarides MCG (2014) Redistribution, inequality, and growth. IMF Staff Discussion Notes International Monetary Fund, Washington DC

Pickett KE, Wilkinson RG (2015) Income inequality and health: a causal review. Social Science and Medicine 128: 316-326

Preston SH (1975) The changing relation between mortality and level of economic development. Population studies 29(2): 231-248

Rajan K, Kennedy J, King L (2013) Is wealthier always healthier in poor countries? The health implications of income, inequality, poverty, and literacy in India. Social Science and Medicine 88: 98-107

Rosero-Bixby L, Dow WH (2009) Surprising SES gradients in mortality, health, and biomarkers in a Latin American population of adults. The Journals of Gerontology Series B: Psychological Sciences and Social Sciences 64(1): 105-117

Sayeed MA, Ali L, Hussain MZ, Rumi MA, Banu A, Khan AA (1997) Effect of socioeconomic risk factors on the difference in prevalence of diabetes between rural and urban populations in Bangladesh. Diabetes Care 20(4): 551-555

Seccareccia F, Pannozzo F, Dima F, Minoprio A, Menditto A, Lo Noce C, Giampaoli, S (2001) Heart rate as a predictor of mortality: the MATISS project. American Journal of Public Health 91(8): 1258-1263

Sherif S, Sumpio BE (2015) Economic development and diabetes prevalence in MENA countries: Egypt and Saudi Arabia comparison. World Journal of Diabetes 6(2): 304

Subramanian SV, Kawachi I (2004) Income inequality and health: what have we learned so far? Epidemiologic Reviews 26(1): 78-91

Wilkinson RG, Pickett KE (2010) The spirit level: why equality is better for everyone. Penguin, London

Xie Y, Zhou X (2014) Income inequality in today’s China. Proceedings of the National Academy of Sciences 111(19): 6928-6933

Xu F, Yin XM, Zhang M, Leslie E, Ware R, Owen, N (2006) Family average income and diagnosed type 2 diabetes in urban and rural residents in regional mainland China. Diabetic Medicine 23(11): 1239-1246

Zhang X, Kanbur R (2005) Spatial inequality in education and health care in China. China Economic Review 16(2): 189-204

Zhao Y, Strauss J, Yang G et al (2014) China health and retirement longitudinal study–2011-2012 national baseline users’ guide.  Peking University, Beijing

Zhu Y (2013) Current approaches to social protection in China. In: Midgley D, Piachuad D (ed) Social Protection, Ecnomic Growth and Social Change. Goals, Issues and Trajectories in China, India, Brazil and South Africa. Edward Elgar, Cheltenham, pp 44-59

*Figure 2:*

(a) Gini Coefficients by province (estimated using CHARLS), China Health and Retirement Survey 2011


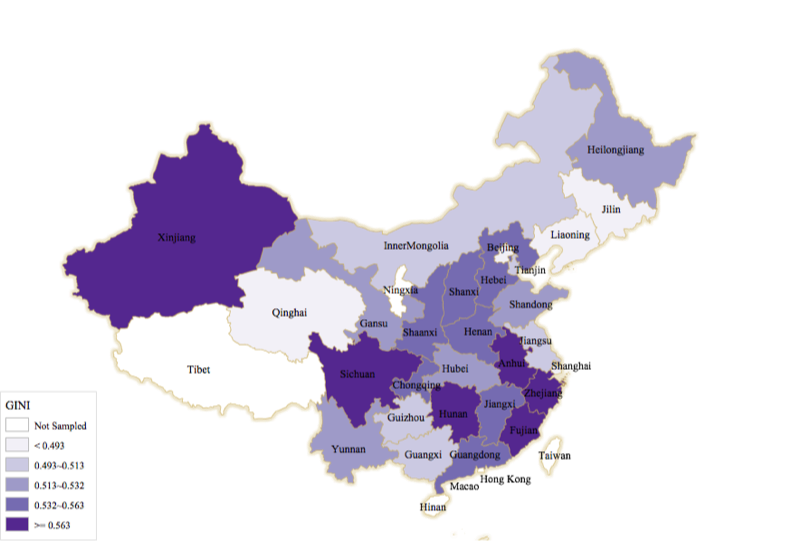


(b) Economic Development (PC_1_) by province, Chinese Statistical Year Book 2012


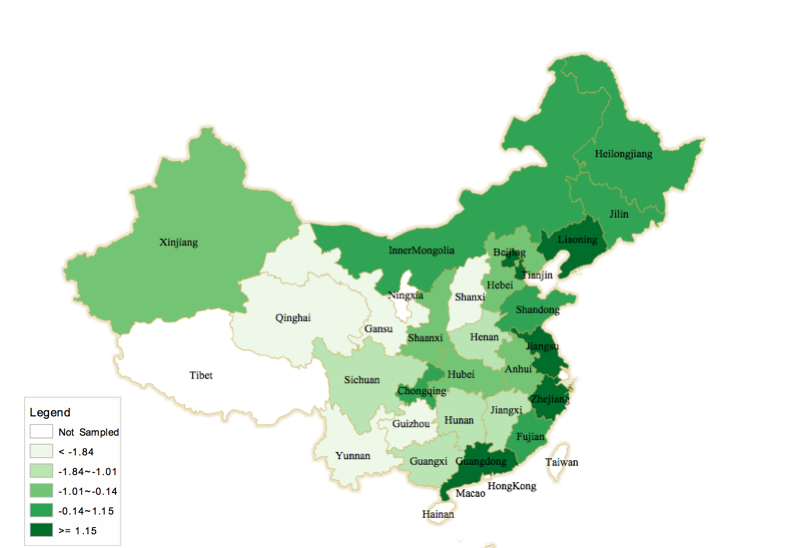


(c) Health-Related Public Infrastructure (PC_2_) by province, Chinese Statistical Year Book 2012


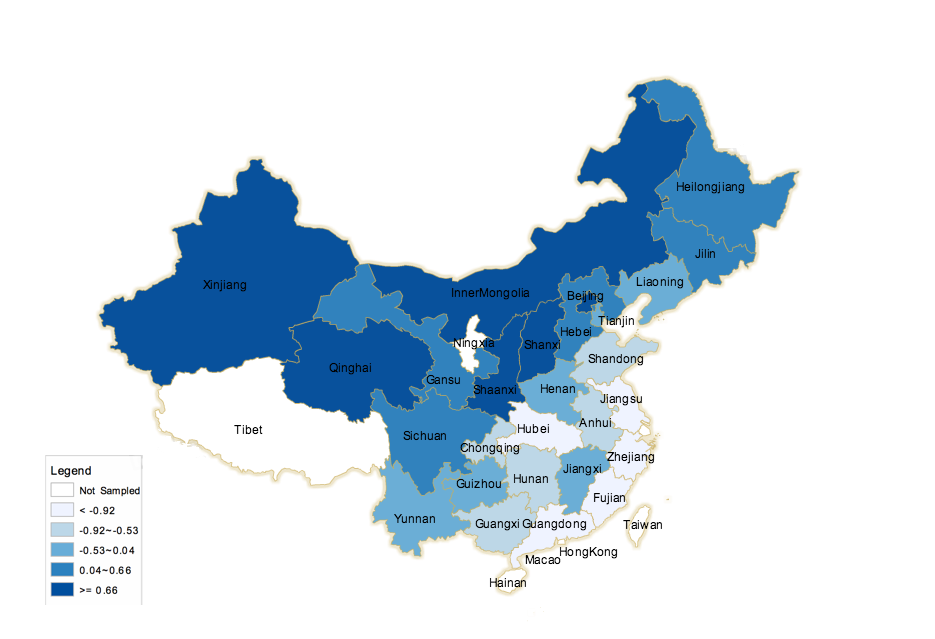


*Table 1:*

| Variable | Cut-off points |
| --- | --- |
| Abdominal girth | Waist > 80cm for women, >90 cm for men |
| Diabetes | Glycosylated hemoglobin $\geq$ 6.5%, or taking medicine |
| High blood pressure | Diastolic > 90 and systolic > 140 mmHg in three measurements, or taking medicine |
| High triglycerides | $\geq$ 50 mg/dl |
| High cholesterol ratio | Total to HDL $\geq$ 5.92 |
| High C-reactive protein | $\geq$ 10mg/L |
| Weak grip strength | <20kg women, 30kg men |
| Allostatic load | Sum of the number of high risk condition (including poor self-rated health) weighted by number of non-missing observations |

Cut-off points for high risk values of individual biomarkers

*Note: Cut-off points used to define “high risk” health conditions*

*Source: Rosero-Bixby & Dow, 2009*

*Table 2:*

Descriptive statistics for health outcomes, China Health and Retirement Survey 2011

| **Health Outcome** | **Sample Size** | **% of Negative Outcomes** | **% of negative outcomes by gender** | | | **% of negative outcomes by age** | | |
| --- | --- | --- | --- | --- | --- | --- | --- | --- |
|  |  |  | **Male** | **Female** | **χ 2** | **45-64** | **65+** | **χ 2** |
| Poor self-rated health | 16,249 | 23.8 | 20.2 | 27.2 | 108.8^***^ | 20.5 | 30.5 | 193.6^***^ |
| Abdominal girth | 12,827 | 48.0 | 28.02 | 66.12 | 1900^***^ | 49.1 | 45.6 | 13.87^***^ |
| Diabetes | 10,893 | 5.4 | 5.0 | 5.8 | 2.9^***^ | 5.0 | 6.2 | 6.77^**^ |
| High blood pressure | 10,319 | 13.0 | 14.0 | 12.3 | 7.0^**^ | 12.5 | 14.3 | 6.5^*^ |
| High triglycerides | 10,808 | 27.3 | 24.6 | 29.6 | 34.0^***^ | 28.8 | 24.1 | 26.4^***^ |
| High cholesterol ratio | 10,802 | 10.0 | 10.0 | 10.1 | 0.1 | 10.1 | 9.7 | 0.6 |
| High C-reactive protein concentrations | 10,815 | 10.3 | 5.2 | 3.7 | 13.6^***^ | 3.7 | 6.0 | 31.5^***^ |
| Weak grip strength | 12,477 | 26.1 | 25.3 | 26.9 | 4.1^*^ | 16.9 | 45.0 | 1100^***^ |
|  |  | **Mean (SD)** | **Mean by gender (SD) T-test** | | | **Mean by age (SD) T-test** | | |
| Allostatic load^a^ | 10,902 | 0.7 (0.5) | 0.6 (0.5) | 0.8 (0.5) | 20.1^***^ | 0.7 (0.5) | 0.8 (0.5) | -8.4^***^ |

*Note: ^*^p < 0.05, ^**^p < 0.01, ^***^p < 0.001; ^a^Allostatic load reported here is log transformed.*

*Table 3:*

Descriptive statistics for individual-level independent variables, China Health and Retirement Survey

2011

| Variables | Mean/Proportion | SD | Min | Max |
| --- | --- | --- | --- | --- |
| Age | 61.01 | 9.81 | 45 | 103 |
| Male | 0.48 | 0.50 | 0 | 1 |
| Marital Status (Married=1) | 0.88 | 0.33 | 0 | 1 |
| Family Size | 3.65 | 1.79 | 1 | 16 |
| Urban Residents | 0.40 | 0.49 | 0 | 1 |
| Illiterate | 0.27 | 0.46 | 0 | 1 |
| Low Education | 0.39 | 0.49 | 0 | 1 |
| Intermediate Education | 0.21 | 0.41 | 0 | 1 |
| High Education | 0.13 | 0.33 | 0 | 1 |
| Household Income (1,000 yuan) | 25.10 | 46.46 | 0 | 2700 |
| Quintile: 1^st^ (1,000 yuan) | 1.16 | 0.87 | 0 | 3 |
| Quintile: 2^nd^ (1,000 yuan) | 6.36 | 2.20 | 30.11 | 30.30 |
| Quintile: 3^rd^ (1,000 yuan) | 15.12 | 3.17 | 10.32 | 20.91 |
| Quintile: 4^th^ (1,000 yuan) | 28.68 | 5.10 | 20.92 | 38.40 |
| Quintile: 5^th^ (1,000 yuan) | 74.47 | 85.45 | 38.40 | 2700 |

*Source: 2011 CHARLS Wave 1 (Baseline)*

*Table 4:*

|  | **Covariates Unadjusted** | | | | **Covariates Adjusted** | | | |
| --- | --- | --- | --- | --- | --- | --- | --- | --- |
| **Estimates** | **Model 1** | **Model 2** | **Model 3** | **Model 4** | **Model 5** | **Model 6** | **Model 7** | **Model 8** |
| ***Provincial-level Explanatory Variables*** | | | | | | | | |
| ED | 0.91^***^ (0.02) |  |  | 0.92^**^ (0.02) | 0.95^*^ (0.02) |  |  | 0.96*^*^* (0.02) |
| Gini Coefficient |  | 1.94 (2.06) |  | 1.63 (1.51) |  | 0.95 (0.91) |  | 1.48 (1.29) |
| HRPI |  |  | 1.09^***^ (0.05) | 1.10^***^ (0.05) |  |  | 1.13^**^(0.05) | 1.14^**^ (0.05) |
| **Individual-level Covariates** | | | | | | | | |
| **Socio-demographic** |  |  |  |  |  |  |  |  |
| Age |  |  |  |  | 1.05^*^(0.02) | 1.05^*^(0.02) | 1.05^*^(0.02) | 1.05^*^(0.02) |
| Male |  |  |  |  | 0.71^***^ (0.03) | 0.72^***^ (0.03) | 0.72^***^ (0.03) | 0.72^***^ (0.03) |
| Married |  |  |  |  | 1.05 (0.06) | 1.04 (0.06) | 1.04 (0.06) | 1.05 (0.06) |
| Household Size |  |  |  |  | 1.02^*^(0.01) | 1.03^*^ (0.01) | 1.03^*^ (0.01) | 1.02^*^(0.01) |
| **Socio-economic Status** |  |  |  |  |  |  |  |  |
| Urban Resident |  |  |  |  | 0.73^***^(0.03) | 0.72^***^(0.03) | 0.72^***^(0.03) | 0.72^***^(0.03) |
| Education (Ref.=Illiterate) |  |  |  |  |  |  |  |  |
| Low Education |  |  |  |  | 0.89^*^ (0.04) | 0.89^*^ (0.04) | 0.89^*^ (0.04) | 0.89^*^ (0.04) |
| Intermediate Education |  |  |  |  | 0.72^***^ (0.05) | 0.71^***^ (0.05) | 0.71^***^ (0.05) | 0.71^***^ (0.05) |
| High Education |  |  |  |  | 0.53^***^ (0.04) | 0.53^***^ (0.04) | 0.52^***^ (0.04) | 0.53^***^ (0.04) |
| Income Quintile (Ref. = 1^st^ ) |  |  |  |  |  |  |  |  |
| 2^nd^ |  |  |  |  | 0.97 (0.05) | 0.97 (0.05) | 0.97 (0.05) | 0.97 (0.05) |
| 3^rd^ |  |  |  |  | 0.79^***^ (0.05) | 0.79^***^ (0.05) | 0.79^***^ (0.05) | 0.79^***^ (0.05) |
| 4^th^ |  |  |  |  | 0.65^***^ (0.04) | 0.65^***^ (0.04) | 0.65^***^ (0.04) | 0.65^***^ (0.04) |
| 5^th^ |  |  |  |  | 0.51^***^(0.04) | 0.51^***^(0.04) | 0.51^***^(0.04) | 0.51^***^(0.04) |
| *ICC* | *0.012* | *0.014* | *0.014* | *0.012* | *0.012* | *0.012* | *0.012* | *0.012* |
| *Level 2 variance* | *0.23^***^(0.04)* | *0.28^***^(0.05)* | *0.26^***^(0.05)* | *0.19^***^ (0.04)* | *0.22^***^(0.04)* | *0.24^***^(0.04)* | *0.20^***^ (0.04)* | *0.18^***^(0.04)* |
| *BIC* | *17697.46* | *17707.18* | *17705.15* | *17713.28* | *17010.4* | *17013.88* | *17006.69* | *17022.37* |

Two-level logit models for self-reported health (poor health=1, odds ratios, N = 16,249), China Health and Retirement Survey 2011

*Note: Robust standard errors are reported in parentheses. Significance levels: † 10%, * 5%, ** 1%, *** 0.1%.*

*Table 5:*

| **Estimates** | **Abdominal Girth** | **Diabetes** | **Hypertension** | **High**  **Triglycerides** | **High**  **Cholesterol**  **ratio** | **High**  **C-reactive**  **Protein**  **concentration** | **Weak grip**  **strength** | **Allostatic**  **Load** |
| --- | --- | --- | --- | --- | --- | --- | --- | --- |
| ***Provincial-level Explanatory Variables*** | | | | | | | | |
| Gini Coefficient | 0.63 (0.94) | 0.32 (0.48) | 0.03^**^ (0.04) | 0.13^*^ (0.14) | 1.46 (1.87) | 1.82 (2.61) | 1.40 (2.13) | -0.66 (0.44) |
| ED | 1.08^*^ (0.04) | 1.02 (0.04) | 0.96 (0.03) | 0.99 (0.03) | 1.06^*^ (0.04) | 1.05 (0.04) | 1.02 (0.04) | 0.01 (0.01) |
| HRPI | 1.12^*^ (0.08) | 1.05 (0.08) | 1.11*^*^* (0.06) | 1.03 (0.06) | 1.02 (0.07) | 1.07^*^ (0.05) | 1.08(0.05) | 0.03^***^ (0.01) |
| **Individual-level Covariates** | | | | | | | | |
| **Socio-demographic** |  |  |  |  |  |  |  |  |
| Age | 1.05^*^(0.02) | 1.28^***^ (0.07) | 1.01^*^ (0.01) | 0.99^**^ (0.01) | 1.00 (0.01) | 1.03^***^(0.01) | 1.08^***^(0.01) | 0.01^***^(0.01) |
| Male | 0.17^***^ (0.01) | 0.77^**^ (0.07) | 1.20^**^ (0.08) | 0.77^***^ (0.04) | 0.99^***^ (0.07) | 1.40^**^ (0.14) | 0.94 (0.05) | -0.34^***^ (0.02) |
| Married | 1.22^**^ (0.08) | 1.41^*^ (0.21) | 0.74^**^ (0.07) | 1.06 (0.08) | 1.18 (0.13) | 1.09 (0.16) | 0.77^***^ (0.05) | -0.01 (0.03) |
| Household Size | 0.97^**^ (0.01) | 0.98 (0.03) | 1.00 (0.02) | 1.00 (0.01) | 1.05^*^ (0.02) | 0.99 (0.83) | 1.03^*^ (0.01) | -0.01 (0.01) |
| **Socio-economic Status** |  |  |  |  |  |  |  |  |
| Urban Resident | 1.72^***^ (0.08) | 1.74^***^ (1.06) | 1.19^**^ (0.08) | 1.32^***^ (0.06) | 1.46^***^(0.10) | 1.07 (0.11) | 0.93 (0.05) | 0.15^***^(0.02) |
| Education (Ref.=Illiterate) |  |  |  |  |  |  |  |  |
| Low Education | 1.10*^†^* (0.06) | 0.97 (0.11) | 0.98 (0.08) | 0.98 (0.06) | 1.12 (0.10) | 0.88 (0.10) | 0.77^***^ (0.04) | -0.01 (0.02) |
| Intermediate Education | 1.21^**^ (0.08) | 1.27*^†^* (0.18) | 0.97 (0.10) | 1.04 (0.08) | 1.13 (0.12) | 0.89 (0.14) | 0.61^***^ (0.05) | -0.02 (0.03) |
| High Education | 1.17^*^ (0.10) | 1.14 (0.19) | 0.88 (0.11) | 0.97 (0.09) | 1.17 (0.15) | 0.62^*^ (0.13) | 0.56^***^ (0.06) | -0.07^*^ (0.03) |
| Income Quintile (Ref. = 1^st^ ) |  |  |  |  |  |  |  |  |
| 2^nd^ | 0.98 (0.06) | 0.93 (0.13) | 0.89 (0.08) | 0.96 (0.07) | 0.94 (0.10) | 0.85 (0.12) | 0.77^***^ (0.05) | -0.05*^†^* (0.03) |
| 3^rd^ | 1.01 (0.07) | 0.99 (0.14) | 0.93 (0.09) | 0.95 (0.07) | 1.03*^†^* (0.07) | 0.73^*^ (0.11) | 0.69^***^ (0.05) | -0.07^*^ (0.03) |
| 4^th^ | 1.10 (0.07) | 0.14 (0.16) | 0.81^*^ (0.08) | 1.01 (0.07) | 1.09^*^ (0.04) | 0.83 (0.13) | 0.62^***^ (0.05) | -0.05*^†^* (0.03) |
| 5^th^ | 1.15 (0.08) | 1.09 (0.16) | 0.88 (0.09) | 1.13 (0.09) | 1.10 (0.10) | 0.85 (0.14) | 0.53^***^(0.04) | -0.05 (0.03) |
| *Intercept* |  |  |  |  |  |  |  | *-0.25^*^ (0.19)* |
| *ICC* | *0.017* | *0.02* | *0.015* | *0.012* | *0.017* | *0.019* | *0.019* | *0.005* |
| *Sample Size* | *12827* | *10893* | *10319* | *10808* | *10802* | *10815* | *12477* | *10902* |

Two-level logit models for single biomarkers (poor health=1, odds ratios), and two-level linear model for allostatic load (Coefficient), China Health and Retirement Suvey 2011

*Note: Robust standard errors are reported in parentheses. Significance levels: † 10%, * 5%, ** 1%, *** 0.1%.*

1. [↑](#footnote-ref-1)
